# American Sign Language Recognition Using Leap Motion Controller with Machine Learning Approach

**DOI:** 10.3390/s18103554

**Published:** 2018-10-19

**Authors:** Teak-Wei Chong, Boon-Giin Lee

**Affiliations:** Department of Electronics Engineering, Keimyung University, Daegu 42601, Korea; chongteakwei@gmail.com

**Keywords:** human-computer interaction, machine learning, sign language recognition, Leap Motion Controller, support vector machine, deep neural network, multi-class classification, American Sign Language

## Abstract

Sign language is intentionally designed to allow deaf and dumb communities to convey messages and to connect with society. Unfortunately, learning and practicing sign language is not common among society; hence, this study developed a sign language recognition prototype using the Leap Motion Controller (LMC). Many existing studies have proposed methods for incomplete sign language recognition, whereas this study aimed for full American Sign Language (ASL) recognition, which consists of 26 letters and 10 digits. Most of the ASL letters are static (no movement), but certain ASL letters are dynamic (they require certain movements). Thus, this study also aimed to extract features from finger and hand motions to differentiate between the static and dynamic gestures. The experimental results revealed that the sign language recognition rates for the 26 letters using a support vector machine (SVM) and a deep neural network (DNN) are 80.30% and 93.81%, respectively. Meanwhile, the recognition rates for a combination of 26 letters and 10 digits are slightly lower, approximately 72.79% for the SVM and 88.79% for the DNN. As a result, the sign language recognition system has great potential for reducing the gap between deaf and dumb communities and others. The proposed prototype could also serve as an interpreter for the deaf and dumb in everyday life in service sectors, such as at the bank or post office.

## 1. Introduction

Communication connects people by allowing them to convey messages to each other, to express their inner feelings, and to exchange thoughts, either verbally or non-verbally. However, deaf community incapable of communicating verbally. As such, the debut of sign language was designed to assist hearing-impaired communities to express their feelings to others. However, sign language is entirely different from spoken language: it has its own grammar and its own manner of expression. Sign language is a non-verbal language that expresses one’s meaning by involving the movement of fingers, hands, arms, head, and body, as well as through facial expressions [1]. Therefore, it can be very challenging for society to learn and practice sign language. This communication barrier has become one of the main reasons that these disability communities (deaf and dumb) are being isolated from society. Fortunately, with the advancement of human–computer interaction technology over the past decades, humans have been able to interact with computers and, in return, receive feedback from the computer. With such methods, many sign recognition systems have been proposed that capture human gestures and then analyze and provide the recognized sign language output, either by text or verbally.

Various research projects have been conducted in recent years regarding sign language recognition. In general, the methods for recognizing sign language can be divided into two general classes: sensor- and vision-based approaches. The sensor-based approach utilizes various sensors that mostly attach to the hands to capture hand gestures. In fact, flex sensors and Inertial Measurement Units (IMUs) are to the two most common sensors for tracking hand gestures [2]. These sensors are used to detect the flexion of fingers and orientation of the hand. Preetham et al. [3] proposed a low-cost data glove with five flex sensors attached to the fingers. The data glove mapped the collected data to a character set for gesture recognition by using a minimum mean square error (MMSE) algorithm. In general, the flex sensor is attached to the finger to measure its bending angle by converting the bendiness into digital output. The higher the degree of the bend in the flex sensor, the higher the value of the digital output. However, the flex sensor is unable to measure the orientation of the finger and hand. Thus, the IMU sensor was adopted to solve this issue. Wang et al. [4] incorporated five unidirectional flex sensors and an IMU into a data glove which classified hand gestures by a template matching algorithm, resulting in a recognition accuracy rate of trained gestures of over 91%. For further improvement, Lee et al. [2] added two pressure sensors to a smart wearable hand device for recognizing 26 American Sign Language (ASL) letters using a support vector machine (SVM). The smart wearable device adopted five flex sensors on the fingers, a three-axis inertial motion on the back of the palm, and two pressure sensors on the middle finger (on the top and on the left, assuming a right-handed glove). The fusion method showed a tremendous improvement in the accuracy rate—from 65.7% to 98.2%—with the introduction of the pressure sensors. Meanwhile, Wu et al. [5] introduced a sensor fusion wearable system for ASL recognition in real time using IMU and surface electromyography (sEMG). An information gain-based feature selection scheme was utilized for the best feature selection subset, and the experimental result showed an accuracy of 96.16% by the SVM classifier. The study revealed that the accuracy rate improved from 3.84% to 15.12% with the addition of the sEMG features using four different classifiers. With a similar approach, Cheng et al. [6] extracted features from an accelerometer and sEMG signals for 223 Chinese Sign Language (CSL) characters with a recognition accuracy of 96%. Both studies indicated that the muscle electrical activity tended to vary according to the different movements of the hand and fingers. In addition, Shukor et al. [7] developed a similar wearable glove with the integration of 10 tilt sensors to measure the fingers’ motion, and an accelerometer was placed on the back of the palm to capture the hand motion. The study reported an average accuracy rate of 89% in ASL recognition. Similarly, Mummadi et al. [8] presented an IMU-based glove equipped with five IMU sensors placed on the fingertips to obtain pitch, roll, and yaw data. The study revealed that the random forest (RF) classifier with 15 subtrees gave the highest accuracy of 92.95% for the recognition of 24 ASL letters. However, sensor-based approaches are often found to be intrusive to the users, as the sensors or data glove can be very bulky and uncomfortable to wear.

Hence, the vision-based approach is an alternative for sign language recognition. This approach utilizes a video camera or visual sensor to capture the movements and analyzes the gestures with certain motion algorithms. Roh et al. [9] found that a dynamic Bayesian network (DBN) model with a five-state hidden node achieved the highest accuracy of 94.6% for 48 ASL signs. Moreover, Bheda et al. [10] presented ASL alphabet and digit recognition separately using a deep convolutional network with an accuracy rate of 67% for the ASL letters and 70% for the digits. The study captured images for each sign and applied background subtraction techniques to remove the background. Meanwhile, instead of background subtraction, Pan et al. [11] presented hand segmentation by extracting skin color in the YCbCr color space determined by applying a Gaussian mixture model (GMM) to find the largest skin blob. YCbCr is a family of color spaces used in video and digital photography systems, where Y, Cb, and Cr represent the luma, blue, and red components, respectively. Overall, this study achieved an accuracy rate of 99.8% for Chinese Sign Language (CSL) recognition and 94% for ASL by an SVM classifier. Beside color spaces, Kang et al. [12] introduced depth sensors to recognize 31 ASL using convolutional neural network with accuracy rate from 83.58% to 85.49% for new signers.

On the other hand, the Microsoft and Leap Motion companies have developed distinct ways to detect and track a user’s hand and body motion by introducing Kinect and the Leap Motion Controller (LMC), respectively. Kinect recognizes the body skeleton and tracks the hands, whereas the LMC only detects and tracks hands with its built-in cameras and infrared sensors [13,14]. Using of the provided framework, Sykora et al. [15] utilized the Kinect system to capture the depth information of 10 hand gestures to classify them using a speeded up robust features (SURF) method that reached up to an 82.8% accuracy rate. Similarly, Huang et al. [16] proposed 10-word-based ASL recognition system using Kinect by 10-fold cross-validation with an SVM that achieved an accuracy rate of 97% using a set of frame-independent features. Meanwhile, Chai et al. [17] also utilized the Kinect system to capture the 3D trajectory of the motion of both hands and the body skeleton to recognize word- and sentence-based CSL with an accuracy rate of 96.32%. Yang et al. [18] proposed a system using Kinect to capture 3D information with a hierarchical conditional random field (CRF) that recognized a 24-word-based sign language. The system revealed an accuracy rate of 90.4%. Khelil et al. [19] developed a recognition system for 28 letters and digits of Arabic Sign Language (ArSL) using the LMC which presented a 91% accuracy rate with an SVM classifier. A similar study was also conducted by Du et al. [20], where 10-digit gestures were recognized using the LMC with an SVM classifier. The study reported that the performance improved significantly, from 83.36% to 98.08%, with the inclusion of histogram of oriented gradient (HOG) features extracted from the LMC sensor data. Additionally, Kumar et al. [21] proposed a recognition system for 20 ArSL signs integrated into one LMC and two digital cameras, resulting in an accuracy rate of 95%. The LMC in the system was used to capture finger movements and the digital cameras were used for capturing body movements and facial expressions. However, Funasaka et al. [22] and Mapari et al. [23] focused on the static (no movements involved) ASL alphabet with the LMC system. Funasaka et al. [22] presented an accuracy rate of 82.71% using an integration of a decision tree and a genetic algorithm, whereas Mapari et al. [23] introduced a 10% hold-out validation multiple layer perceptron (MLP) with a 90% accuracy rate. Recognition of ASL signs that involve movement for certain letters can be challenging, and several studies, such as those by [22,23,24], were only able to detect the ASL alphabet when the dynamic ASL signs for the letters ’J’ and ’Z’ were excluded.

**Figure 1:**
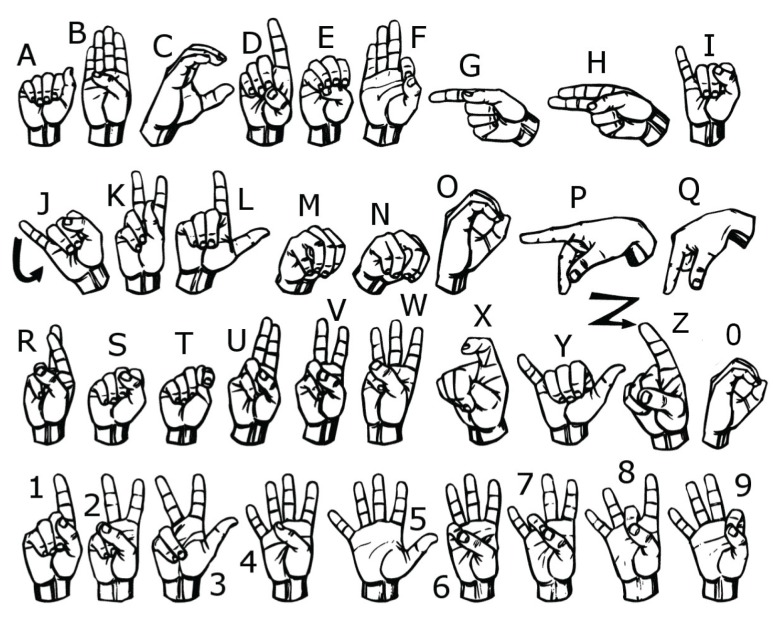
The 26 letters and 10 digits of American Sign Language (ASL).

However, sign language is not standardized globally, similar to the fact that each country has its own language. Some examples of sign languages are ASL, ArSL, Greek Sign Language (GSL), CSL, Korean Sign Language (KSL), etc. [1]. The aimed contribution of this study was to develop an ASL recognition system using the LMC system that involves complete sets of 26 letters (A–Z) and 10 digits (0–9) of ASL (depicted in Figure 1); such a system is not found in other existing studies. The extracted features served as inputs to two different classifiers—namely, a support vector machine (SVM) and deep neural network (DNN)—and the predicted results were compared for their accuracy rates. There are 36 classes of ASL fingerspelling recognition utilized in this study. In fact, ASL fingerspelling recognition is an important milestone in facilitating communication between two communities, as ASL is not a code for English [25]. ASL is an independent language which use signs to convey a message, and fingerspelling is used to spell out a name of anything lacking a specific corresponding sign [10].

This paper is organized into five sections. Background research and the most recent related studies are presented in the first section to give the readers clear contextual and background information of the topic. The next section is the proposed system overview and details of the LMC. Next, Section 3 presents four subsections: data collection, feature extraction, feature preprocessing, and classification. In Section 4, the classification results and discussion are presented. The discussion of each ASL letter and digit pair correlation are discussed as well, and, lastly, the paper ends with a summary of the proposed study.

## 2. System Overview

**Figure 2:**
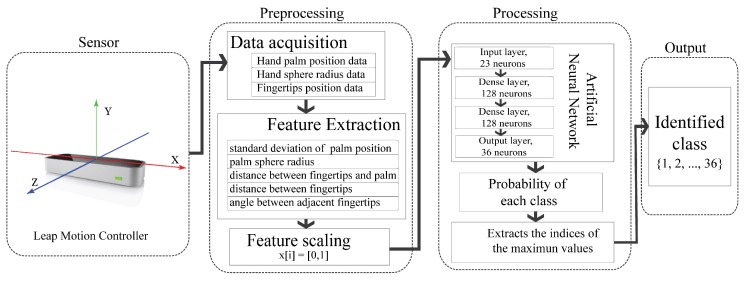
Proposed system flow used in the study.

Nowadays, the LMC is widely used in different areas, such as gaming, device control, interactive art, virtual reality, and other fields. The LMC is a low-cost and palm-sized portable peripheral device which is specifically designed to track hand and finger motion with high precision in the 3D Cartesian coordinate system, as illustrated in the sensor module of Figure 2. The device consists of two monochromatic cameras and three infrared light-emitting diodes (LEDs) [13] and has an interaction field of approximately 8 cubic feet above the device. The LMC is provided with an application programming interface (API) which allows users to obtain data on the hand and fingers, e.g., fingertip position, hand palm position, etc. [13]. Unfortunately, not all the provided information is useful and suitable for sign language recognition. For instance, the absolute hand and finger positions are not quite relevant for gesture detection, but integration with other information can provide more meaningful information for sign language recognition [19].

The proposed system in this study consists of four modules, namely, the sensor, preprocessing, processing, and output modules, as shown in Figure 2. In the sensor module, the LMC is used for data acquisition, which includes a three-axis hand palm position, the hand sphere radius, and the position of the five fingertips. All collected data are subjected to the preprocessing module in order to extract 23 meaningful features. Lastly, the processing module provides the classification results in the range of 1–36, where the outputs 1–26 represent the letters from ‘A’ to ‘Z’, and the outputs 27–36 represent the digits from ‘0’ to ‘9’. Generally, this study utilized two classifiers, SVM and DNN, for comparison purposes. Detailed discussions are presented in Section 3, below.

## 3. Method

### 3.1. Data Collection

**Figure 3:**
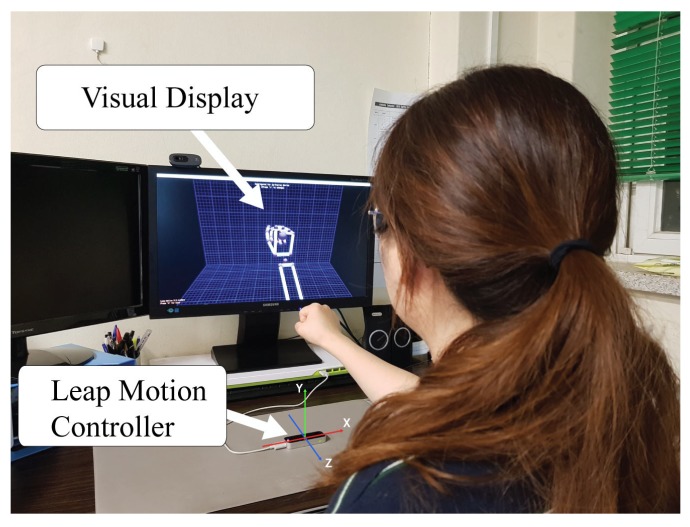
Experimental setup with a real-time hand gesture in 3D graphic display.

In order to collect the necessary data, 12 voluntary subjects were recruited from the university to participate in the experiments. It is clarified that the current study is the initial study; thus, the focus subject group did not include deaf or dumb individuals or experts in sign language, though they will definitely be included for future work. Before the experiment, the subjects were given approximately 1 h to become familiar with ASL as well as the LMC operating method before the real experiment was conducted. Once the subjects were confidently familiar with the prototype system and the sign language, experiments were conducted as illustrated in Figure 3. The LMC device was connected to a desktop PC and placed on the table to detect and track the subject’s hand and finger gestures. The monitor displayed the 3D model of the subject’s current gesture. The subject was requested to perform all ASL gestures, including the 26 letters and 10 digits. A laboratory member with experience in ASL sat beside the subject to assist and verify the correctness of his/her hand gestures throughout the experiments. All data collection was completed within 10 s for each sign at a 60 Hz sampling rate. The duration for performing each sign was slightly different depending on the complexity of the sign. However, during the recording phase, the subjects were requested to hold the gestures, except for the letters ‘J’ and ‘Z’, which require movement.

### 3.2. Feature Extraction

**Figure 4:**
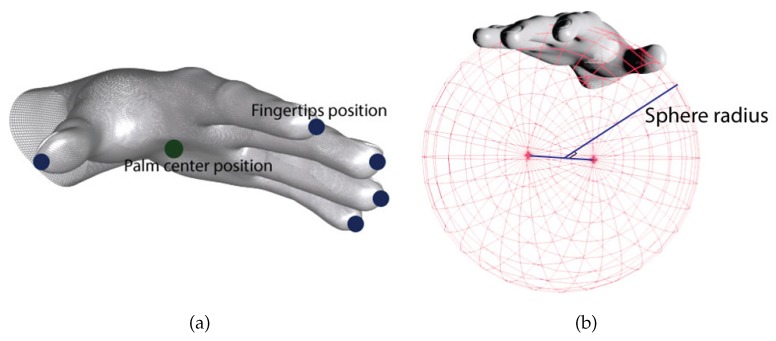
The position of (**a**) palm center and fingertips illustrated in (**b**) sphere radius mode.

In this study, the adopted basic features included the hand palm sphere radius, the hand palm position, and fingertip position, as depicted in Figure 4a. The hand palm sphere radius *R* measures the radius of a sphere fit to the curvature of the hand [13], as illustrated in Figure 4b, where the hand is imagined as holding a virtual sphere, with the radius of the sphere defined by the degree of curliness of the fingers curled into a fist. The two red dots illustrated in Figure 4b are the top and bottom points of the sphere, and the line connecting the two red dots is the diameter of the sphere; thus, half of the diameter is the radius of the sphere, the so-called “hand palm sphere radius”. The palm position *P* represents the absolute 3D position of the palm center, whereas the fingertip positions $F_{i}$ are the positions of the fingertips, with *i* = 1, …, 5 representing the index of the fingers in order of thumb, index finger, middle finger, ring finger, and pinky finger, respectively. Subsequently, given the aforementioned data, features were extracted and organized into five distinct groups, known as S, R, D, A, and L, as shown in Table 1. Group S features represent the standard deviation of the absolute 3D (*x*-, *y*-, and *z*- coordinates) palm positions, computed as depicted in Equation (1),

(1) $$S=\sqrt{\frac{1}{n-1}\left(\sum_{i=1}^{n}{\left(P_{i}-P\right)}^{2}\right)}$$

where *n* represents the size of the dataset. *n* is defined as 20, which allows the algorithm to perform three calculations per second. Meanwhile, group R features denote the palm sphere radius, whereas group D features are the values of the 3D Euclidean distances of the palm center to each of the fingertips, calculated as shown in Equation (2).

(2) $$D_{FP}=\sqrt{{\left(F_{i_{x}}-P_{x}\right)}^{2}+{\left(F_{i_{y}}-P_{y}\right)}^{2}+{\left(F_{i_{z}}-P_{z}\right)}^{2}}$$

Group A features represent the angles between two adjacent fingers, i.e., the angles between the thumb fingertip and index fingertip, index fingertip and middle fingertip, middle fingertip and ring fingertip, and ring fingertip and pinky fingertip. These four features were computed as Equation (3). However, the angle between the thumb fingertip and pinky fingertip was excluded because this angle includes palm curliness, which is included in feature R.

(3) $$Aa_{F_{i}}=∠\frac{F_{i}-F_{i+1}}{\pi }$$

Lastly, group L features indicate the distance between one fingertip and the consecutive fingertips, e.g., the distance between thumb and index finger, thumb and middle finger, thumb and ring finger, thumb and pinky finger, index finger and middle finger, and so on and so forth. There are 10 features in this feature group, and all of these features were calculated similarly by the 3D Euclidean method, as in Equation (4),

(4) $$L_{FF}=\sqrt{{\left(F_{i_{x}}-F_{j_{x}}\right)}^{2}+{\left(F_{i_{y}}-F_{j_{y}}\right)}^{2}+{\left(F_{i_{z}}-F_{j_{z}}\right)}^{2}}$$

where *i* and *j* = 1, …, 5 represent all five fingertips, while *i* ≠ *j* means there is no distance calculated between a given fingertip and itself. Overall, a total of 23 features were extracted and served as input parameters to the classifiers. All 36 ASL signs have different gesture representations of hand and finger shape; that is, these 23 features were targeted to represent the hand movement, palm flexion, relation of palm and fingertips, and relation of fingertips to fingertips. For instance, feature group S is targeted to represent the hand movement, feature group R is targeted to describe the flexion of the palm, and feature group D represents the relation of the palm and fingertips. Lastly, feature group A and L are targeted to describe the relation of all fingertips for all 36 ASL signs.

### 3.3. Features Preprocessing

In order to reduce the processing time and complexity, all the features were normalized in the range [0, 1]. Due to the differences of each subject’s palm size and finger lengths, all the related features were further scaled on a subject-by-subject basis, where the features were scaled as shown in Equation (5),

(5) $$x=x/\max(x_{s})$$

where *s* represents the maximum value of the features of each individual subject. To further study the implication of each feature group to ASL recognition, the features were divided into six groups, with the combinations shown below:

1. C1: D + A + R + L (S excluded)
2. C2: S + A + R + L (D excluded)
3. C3: S + D + R + L (A excluded)
4. C4: S + D + A + L (R excluded)
5. C5: S + D + A + R (L excluded)
6. C6: S + D + A + R + L (all included)

Categories 1–5 are a unique combination of four feature groups, and the last category includes all the features as inputs to the classifier.

### 3.4. Classification and Validation

In this study, SVM and DNN classifiers were compared for ASL recognition. The training dataset is denoted as Equation (6),

(6) $$\left(x{}_{j}{}^{i},y_{j}\right),y_{j}\in \left\{0,\ldots ,35\right\}$$

where *x* and *y* represent the *i*-th feature and label for *j*-th training dataset, respectively. However, an SVM is a binary classifier, where the output is either −1 (negative) or 1 (positive). The decision boundary of the SVM is also known as a separating hyperplane. Specifically, an SVM with a linear kernel function was utilized in this study, and the separating hyperplane is defined in Equation (7),

(7) f(x)=wx−b

where the *j*-th data is classified as 1 (positive) if f(x)>0, and −1 (negative) if f(x)<0. The data points used to generate the separating hyperplane are known as support vectors, and the nearest distance between the hyperplane and the support vector is known as a margin. Thus, Equation (8) shows the formula of finding the smallest margin among all support vectors.

(8) $$\min_{w,b}=\frac{{∥w∥}^{2}}{2}$$

However, given the multi-classes problem with a total of 36 classes (26 letters and 10 digits) in this study, a multi-class SVM was implemented. As such, by utilizing the Scikit-learn machine learning framework library [26], the decision function was modified by implementing the “one-vs-rest” (OVR) decision method. OVR is also known as “one-vs-all” (OVA) or “one-against-all”, and treats a class (class to observed) as a positive label and the rest of the classes as negative labels. The process is repeated with each class treated as a positive label exactly once, for a total of n times round, where n is the total of classes (36 in this case). Then, the results are integrated for a multi-class classification according to the highest score of each binary classifier $x_{j}$, where *x* represents the confidence value (score) and *j* refers to the designated classifier. Hence, *x* is classified according to the class of the highest confidence value. On the other hand, DNN was implemented in the TensorFlow machine learning framework with the high-level Keras API [27]. The adopted neural network consisted of two hidden layers, with 128 neurons for each layer. All neurons are densely connected using the rectified linear unit (ReLU) activation function to process the data before proceeding to the following layer. The ReLU activation function is computed as Equation (9) below:

(9) f(x)=max(0,x)

where the output is 0 if the input is less than 0, while the output is the raw input if the input is greater than 0. Recently, ReLU has been widely used for the hidden layers in deep learning networks. Meanwhile, the last layer is computed as 36 neurons (26 in cases without digits) with the Softmax activation function. The Softmax activation function returns an array of 36 probability scores with the sum total of 1, as shown in Equation (10),

(10) $$\sigma {\left(z\right)}_{j}=\frac{e_{j}^{z}}{\sum_{k=1}^{K}e_{k}^{z}}$$

where *z* is the input vector from the previous layer; *j* indexes the output units from 1 to *k*; and *k* = 26/36 in this study. Each output nodes contain the probability of the input instance belonging to one of the 26/36 classes. Lastly, all DNN layers were subjected to compilation using the adaptive moment estimation (Adam) optimizer and sparse categorical cross-entropy loss metric into a classification model for training and testing. The Adam optimizer by TensorFlow was set to a learning rate of 0.001, exponential decay rate for first-moment estimates of 0.9, exponential decay rate for second-moment estimates of 0.999, and the very small number of 1e−8 was used to prevent any division by zero in the implementation. The sparse categorical cross-entropy loss metric is used for the classification task to calculate the error, as the neural network aims to minimize loss.

The leave-one-subject-out (LOO) approach was utilized for dataset training and testing, where the k−1 subjects’ dataset formed the training dataset, and the leftover subjects’ dataset was treated as the testing dataset. The process was repeated, with each subject dataset used for testing exactly one time. In another word, this approach trained and tested from different combinations of subjects’ datasets accordingly, and there was no crossing between testing data from the same subject at any validation loop. This subject-oriented approach avoided subject bias successfully. Based on the study by Arlot et al. [28], LOO is the best approach for classifier model training and testing, where the testing dataset not participating in the training process can derive high-confidence classification results even with new data from other users that were not in the training dataset. Moreover, the LOO cross-validation approach has been used in several sign language recognition studies [24,29,30].

## 4. Result and Discussion

In order to determine the flexibility and credibility of the trained classifier, the accuracies (ACC) of the classifiers were computed, as shown in Equation (11):

(11) $$ACC=\frac{TP+TN}{TP+TN+FP+FN}$$

where TP, TN, FP, and FN denote true positive (correctly identified), true negative (correctly rejected), false positive (incorrectly identified), and false negative (incorrectly rejected), respectively. Table 2 illustrates the accuracy results of the trained model for the SVM and DNN classifiers by applying six distinct feature groups as the training dataset. The preliminary result showed that the DNN significantly outperformed the SVM in both the 26-class (90.58% vs. 75.5%) and 36-class (85.65% vs. 67.54%) ASL recognition. Overall, the feature group that, on average, performed best as the training dataset, as determined by accuracy rate, is group C6 with a mean accuracy rate of 83.78%, followed by C4 with a mean accuracy of 83.25%, C2 with a mean accuracy rate of 80.45%, C3 with a mean accuracy rate of 78.95%, C1 with a mean accuracy rate of 78.31%, and last, with the lowest accuracy, is C5 with a mean accuracy rate of 74.17%. This implies that group L makes the most significant contribution to the ASL recognition system among all other features. C5 had the lowest mean accuracy rate, resulting in group L’s exclusion as a training dataset. Moreover, the results also indicated that the DNN classifier had the best performance with the C6 feature group at 93.81% and 88.79% for the 26 classes (26 letters only) and 36 classes (26 letters and 10 digits), respectively. However, the SVM classifier presented distinct results: C4 actually performed better than C6 as the training dataset for the 26 classes (C4 accuracy of 80.30% vs. C6 accuracy of 79.73%). However, C6 was still the best-performing feature group for the 36 classes with the SVM at an accuracy rate of 72.79% compared to the other feature groups. Overall, the DNN had the best ASL recognition performance using the C6 feature group for both the 26 and 36 classes.

**Figure 5:**
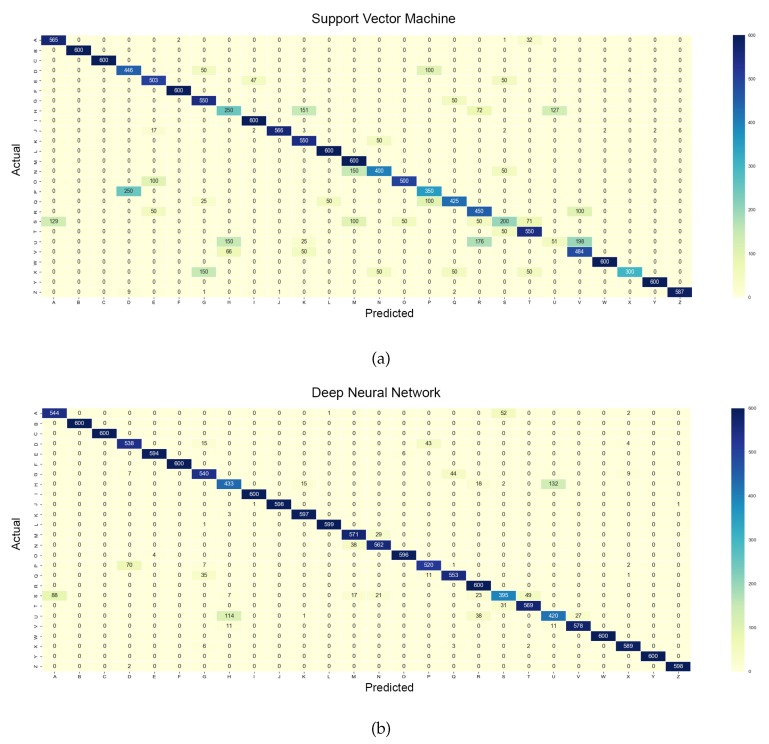
Confusion matrices of 26 classes. (**a**) Support vector machine. (**b**) Deep neural network.

**Figure 6:**
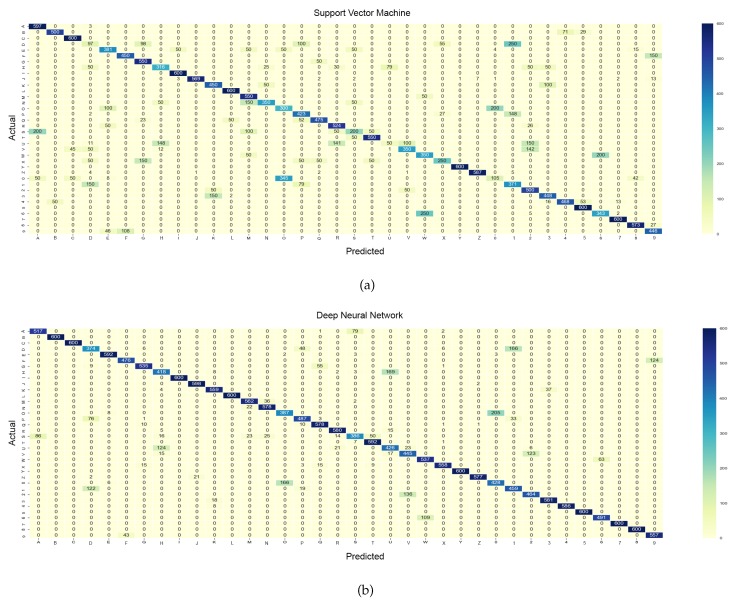
Confusion matrices of 36 classes. (**a**) Support vector machine. (**b**) Deep neural network.

In addition, Figure 5 and Figure 6 present the confusion matrices for the 26 classes and 36 classes, respectively, for both classifiers. To further investigate the true classification rate of each letter and digit, the sensitivity and specificity of the classes were calculated and are presented in Table 3. Sensitivity (Se), also known as the true positive rate, represents the percentage ability of the classifier to correctly identify the actual class. On the other hand, specificity (Sp), also known as the true negative rate, shows the ability of the classifier to correctly reject an instance which is not an actual class. Equations (12) and (13) below show the computation of sensitivity and specificity, respectively.

(12) $$Se=\frac{TP}{TP+FN}$$

(13) $$Sp=\frac{TN}{TN+FP}$$

In terms of the 26 classes (see Figure 5), the letters ‘B’, ‘C’, ‘F’, ‘I’, ‘W’, and ‘Y’ had the highest sensitivities, i.e., 100% (highest confidence level), which indicates that those classes were perfectly classified by both the classifiers. The letters ‘L’ and ‘M’ had 100% sensitivities with the SVM classifier but only 99.83% for ‘L’ and 95.17% for ‘M’ with DNN. On the contrary, the letters ‘H’, ‘S’, and ‘U’ were found to have the lowest confidence levels. Specifically, with the SVM classifier, the letter ‘U’ had the lowest sensitivity (8.5%), followed by the letters ‘S’ (33.33%), ‘H’ (41.67%), and ‘X’ (50%). The letter ‘U’ was incorrectly identified as the letter ‘V’ (33%), ‘R’ (29%), and ‘H’ (25%), according to Figure 5a. Conversely, with the DNN classifier, the letter ‘S’ had the lowest sensitivity percentage of 65.83%, followed by the letters ‘U’ (70%) and ‘H’ (72.17%). The letter ‘S’ was misclassified as the letter ‘A’ (14.67%), ‘H’ (1.17%), ‘M’ (2.83%), ‘N’ (3.5%), ‘R’ (3.83%), and ‘T’ (8.17%), as shown in Figure 5b. Overall, the letters ‘U’ and ‘H’ were reported as the lowest confidence level alphabets in both the SVM and DNN classifiers. Both letters were mutually misclassified as each other and also easily misclassified as the letter ‘R’. The main reason could be that the letters ‘H’ and ‘U’ are highly similarity in term of finger gestures; the only difference is the orientation of the hand.

On the other hand, Figure 6 depicts the confusion matrix for 36 classes. The results indicate that the letters ‘C’, ‘I’, ‘L’, and ‘Y’ and the digits ‘5’ and ‘7’ had a perfect classification rate (100%). Nevertheless, there are several letters and digits that had sensitivity levels below 50% with the SVM classifier: the letters ‘D’ (16.17%), ‘O’ (50%), ‘U’ (8.33%), and ‘X’ (41.67%), and the digit ‘0’ (17.5%). Meanwhile, in terms of the DNN classifier, the letters ‘D’ (62.33%), ‘H’ (69.67%), ‘O’ (64.5%), ‘S’ (64.33%), ‘U’ (71%), and ‘V’ (74.17%), and the digit ‘0’ (71.33%) are the letters and digit with sensitivity levels below 75%. By referring to Table 3, the letters ‘D’, ‘O’, and ‘U’ and the digit ‘0’ had the lowest sensitivity percentage in both classifiers. In general, the letters ‘U’, ‘H’, and ‘R’ were often found to be mutually misclassified with both classifiers. Also, the letter ‘D’ was found to be misclassified as the digit ‘1’ in both classifiers with percentages of 41.67% and 27.67% for the SVM and DNN, respectively. Moreover, the letter ‘O’ and the digit ‘0’ were significantly correlated, as both have similar gestures, and were mutually misclassified with mean accuracy rates of 45.62% and 30.92% for the SVM and DNN, respectively.

Similarly, for the 36 classes with the DNN, the letters ‘B’, ‘C’, ‘I’, ‘L’, and ‘Y’ and the digits ‘5’, ‘7’, and ‘8’ had the highest specificity (100%) and the letter ‘D’ and digit ‘0’had the lowest (99%). Meanwhile, of the 36 classes with the SVM classifier, none had 100% specificity, but the lowest specificity was obtained for the digits ‘2’ (98.22%) and the highest specificity was obtained for the letters ‘J’ (99.99%) and ‘Y’ (99.99%). In the case of the 26 classes, the letters ‘B’ and ‘C’ had perfect specificity with both classifiers for the 26 classes. Further, the letters ‘F’, ‘W’, and ‘Y’ had 100% specificity with the DNN and 99.99% in the SVM. The lowest true negative rate with both classifiers was attained with the letter ‘A’: 99.14% for the SVM and 99.41% for the DNN. Generally, this indicates that both classifiers generally performed well in rejecting the non-classes correctly.

**Figure 7:**
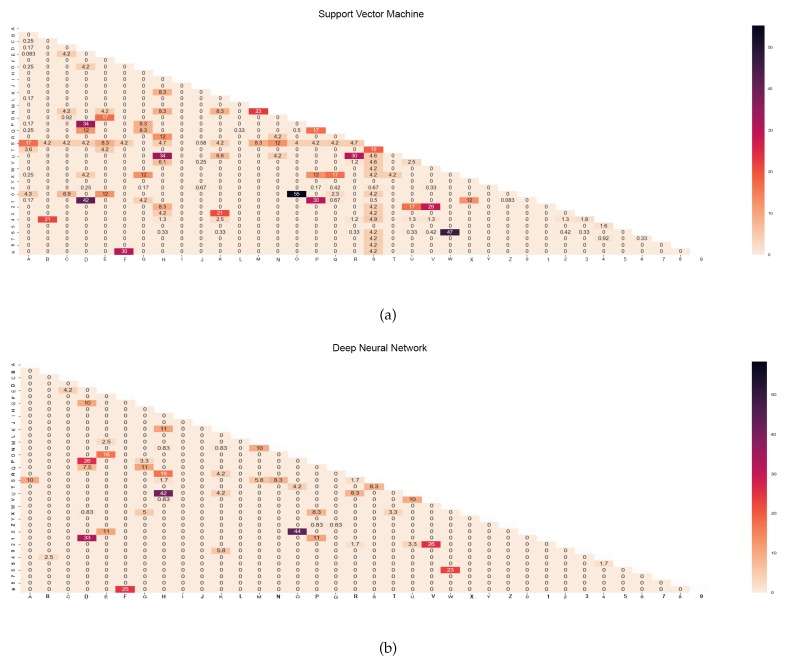
Class-to-class similarity rate matrix for both classifiers (%). (**a**) Support Vector Machine. (**b**) Deep Neural Network.

Class-to-class comparison was performed in order to further investigate the similarities between gestures. Figure 7 depicts the correlation (similarity percentage) of each ASL letter pairwise for both the SVM and DNN classifiers. A high correlation percentage between two letters signifies that both letters showed high similarity in terms of gestures. To the contrary, a low correlation value between two letters depicts significant differences between the two letters. Similar to the previous results, the paired letters ‘H’ and ‘U’ had relatively higher correlation than the others, with values of 34% and 42% for the SVM and DNN classifiers, respectively. Also, the letters ‘H’ and ‘R’ had correlation percentages of 12% with the SVM classifier and 18% with the DNN classifier. These three letters have similar gestures, with the index and middle fingers fully open and the other fingers fully bent (see Figure 1). The only significant difference is the fingers’ pointing direction and the placement of the index finger under the middle finger for the letter ‘R’. In addition, there were also several significant similarities found in the letter-to-digit comparison for both classifiers: ‘O’-‘0’; ‘D’-‘1’; ‘W’-‘6’; ‘F’-‘9’. Among these pairs, ‘O’-‘0’ had the most significant similarity percentage: 55% and 44% with the SVM and DNN classifiers, respectively. This is due to the letter ‘O’ and digit ‘0’ having the exact same finger gestures, where the only difference is the hand orientation (finger pointing direction). The digit ‘1’ also exhibited high similarity with the letter ‘D’ (SVM: 42%; DNN: 33%) as well as with the letter ‘P’ (SVM: 30%; DNN: 11%). The only difference between the digit ‘1’ and the letter ‘D’ is that the thumb is placed under the index finger in the letter ‘D’, rather than overlapped on the index finger as it is in the digit ‘1’. Also, both gestures had exactly the same gesture, with a minor difference in the bendiness of the pinky finger (see Figure 1). Moreover, the digit ‘1’ and the letter ‘P’ differ in the index finger bendiness, but the hand orientation and finger depth information cannot be obtained with the LMC in its current state; thus, they look alike from the LMC data. The letter ‘F’ and digit ‘9’ had an average similarity percentage of 30% for the SVM and 25% for the DNN; these signs are only differentiated by the distance between the index and middle fingers as well as between the middle and ring fingers. The digit ‘2’ is also highly similar to the letter ‘V’: the index and middle fingers for the letter ‘V’ are not bent (slightly bent for the digit ‘2’), but the distances between these fingers are slightly different, with a larger distance for the digit ‘2’ compared to the letter ‘V’. Likewise, the letter ‘W’ and the digit ‘6’ had a similarity percentage of 47% with the SVM and 23% with the DNN classifiers. These two signs are only distinguishable by the small difference in the location of the pinky finger and the slight difference in the hand orientation, as letter ‘W’ is oriented to the right by several degrees.

Last, but not least, Table 4 illustrates the comparison of our proposed methods with the systems and methods conducted by other researchers. It was observed that the most common classifier applied is the SVM classifier [19,20,24,31]. Du et al. [20] reported the highest accuracy rate (99.42%) with the SVM classifier but only with the recognition system of 10 digits (no letters involved). Likewise, a similar method was also employed by Khelil et al. [19], with an accuracy rate of 91.3%. Funasaka et al. [22] and Marin et al. [24] proposed an incomplete ASL recognition system that only recognized 24 letters, with letters ‘J’ and ‘Z’ excluded from the system, and they reported an accuracy rate of 82.7% (using a decision tree) and 80.86% (using an SVM classifier), respectively. On the other hand, an ASL recognition system of 32 letters and digits which did not include ‘J’, ‘Z’, ‘2’, and ‘6’ was proposed by Mapari et al. [23] by utilizing an MLP classifier, which resulted in an accuracy rate of 90%. In fact, even though other existing studies had better-performing ASL recognition rates, those studies did not include all 26 letters and 10 digits (complete ASL set) in their studies. However, our proposed methods utilized all ASL letters and digits, and the systems resulted in mean accuracy rate, sensitivity, and specificity values of over 90%.

## 5. Conclusions

This paper presents an American Sign Language recognition system which involves 26 letters and 10 digits using the Leap Motion Controller. A total of 23 features were adopted in the study and further divided into six different groups of combinations. The results indicate that the distance between one fingertip and the adjacent fingertips (group L) is a significant feature for sign language recognition. Generally, the DNN classifier outperformed the SVM classifier for both the 26-class and 36-class ASL recognition systems. Also, the sign language recognition system with the digits included presented a lower mean accuracy rate due to the large similarity between certain letters and digits. Moreover, even though the accuracy rate obtained in this study is lower compared to existing studies, our study included all ASL letters and digits, whereas other studies did not. There are several issues to be considered in future work, such as the methods to measure the finger and hand depth information, computation of the finger and hand orientation, and detection of overlapping between fingers. Other future works should also consider expanding the sign language recognition system to word- and sentence-based recognition, as well as to other languages, instead of limiting recognition to only ASL.

## Figures and Tables

**Table 1:** Feature group organization.

Group	Feature	# of Feature
S	Standard deviation of palm position	3
R	Hand palm curvature radius	1
D	Distance between palm center and each fingertips	5
A	Angle between two adjacent fingertips	4
L	Distance between one fingertip and the consecutive fingertip	10

**Table 2:** Overall accuracy of 26 classes and 36 classes.

	Accuracy (%)				
Combination	26 Classes		36 Classes		-
	SVM	DNN	SVM	DNN	Average
C1	74.26	87.84	67.85	83.29	74.31
C2	74.57	92.77	67.08	87.38	80.45
C3	75.35	88.61	67.94	83.89	78.95
C4	80.30	93.29	72.04	87.35	83.25
C5	68.81	87.15	57.53	83.18	74.17
C6	79.73	93.81	72.79	88.79	83.78
Average	75.50	90.58	67.54	85.65	-

**Table 3:** Sensitivity (Se) and specificity (Sp) of the ASL letters for 26 classes and 36 classes for both  classifiers.

	36 Classes				26 Classes			
Letters	SVM		DNN		SVM		DNN	
	Se (%)	Sp (%)	Se (%)	Sp (%)	Se (%)	Sp (%)	Se (%)	Sp (%)
A	99.50	98.81	86.31	99.59	94.17	99.14	90.82	99.41
B	83.33	99.76	100.00	100.00	100.00	100.00	100.00	100.00
C	100.00	99.55	100.00	100.00	100.00	100.00	100.00	100.00
D	16.17	99.48	62.33	99.00	74.33	99.32	89.67	99.81
E	63.50	99.03	98.67	99.93	83.83	99.58	99.00	99.99
F	75.00	99.49	79.33	99.80	100.00	99.99	100.00	100.00
G	91.67	98.71	89.17	99.85	91.67	99.58	90.00	99.89
H	52.67	99.00	69.67	99.22	41.67	99.59	72.17	99.77
I	100.00	99.75	100.00	100.00	100.00	99.91	100.00	99.99
J	94.83	99.99	99.67	99.90	94.33	99.99	99.67	10.00
K	75.00	99.05	93.17	99.84	91.67	99.55	99.50	99.97
L	100.00	99.75	100.00	100.00	100.00	99.90	99.83	99.99
M	91.67	98.33	96.67	99.79	100.00	99.50	95.17	99.90
N	58.33	99.64	96.33	99.71	66.67	99.80	93.67	99.91
O	50.00	98.12	64.50	99.20	83.33	99.90	99.33	99.99
P	70.50	98.66	81.17	99.62	58.33	99.58	86.67	99.90
Q	79.17	99.50	96.33	99.65	70.83	99.79	92.17	99.91
R	87.33	98.95	96.67	99.82	75.00	99.37	100.00	99.85
S	33.33	99.28	64.33	99.55	33.33	99.67	65.83	99.83
T	91.67	99.52	98.67	99.72	91.67	99.67	94.83	99.90
U	8.33	99.62	71.00	99.04	8.50	99.72	70.00	99.72
V	58.33	99.28	74.17	99.24	80.67	99.34	96.33	99.95
W	58.33	98.57	89.50	99.48	100.00	99.99	100.00	100.00
X	41.67	99.61	93.00	99.95	50.00	99.99	98.17	99.96
Y	100.00	99.99	100.00	100.00	100.00	99.99	100.00	100.00
Z	97.83	99.97	96.17	99.99	97.83	99.99	99.67	99.99
0	17.50	99.02	71.33	99.00	-	-	-	-
1	61.83	98.08	76.50	99.05	-	-	-	-
2	83.33	98.22	77.33	99.39	-	-	-	-
3	74.67	99.21	96.83	99.80	-	-	-	-
4	78.00	99.66	97.67	99.99	-	-	-	-
5	100.00	99.61	100.00	100.00	-	-	-	-
6	57.17	99.05	81.83	99.70	-	-	-	-
7	100.00	99.92	100.00	100.00	-	-	-	-
8	95.50	99.73	100.00	100.00	-	-	-	-
9	74.33	99.10	92.83	99.41	-	-	-	-

**Table 4:** Comparison of sign language recognition systems using the Leap Motion Controller.

Author	Gesture	Dataset (M people × N repetitions per letter per person)	Cross-Validation	Features	Classifier	Accuracy (%)
Khelil et al. [19]	10 ArSL digits gesture	10 people × 10 sets	-	Angle between two fingertips, angle between fingertips and hand’s normal, distance between the hand center to each fingertips	SVM	91.3
Du et al. [20]	10 digits gesture	13 people × 20 sets	80% training set, and 20% testing set, experiment performed 50 times	Fingertips angle (A), fingertips distance (D), fingertips elevation(E), fingertips tip distance (T)	SVM	83.36
				A+D+E+T+ HOG	SVM	99.42
Funasaka et al. [22]	24 ASL gestures (static)	-	-	Palm normal vector, fingertips position, arm direction and fingertips direction	Decision Tree	82.7
Marin et al. [24]	10 ASL gestures	14 people × 10 sets	Leave-one-subject-out cross-validation	Fingertips angle fingertips distance, and fingertips elevation	SVM	80.86
Chuan et al. [31]	26 ASL gestures	2 people × 2 sets	4 fold cross-validation	Pinch strength, grab strength, average distance, average spread, average tri-spread, extended distance, dip-tip projection, OrderX, and angle	k-Nearest Neighbors SVM	72.78 79.83
Mapari et al. [23]	32 ASL gestures (J, Z, 2 and 6 are excluded)	146 people × 1 set	90% training set, and 10% testing set cross-validation	Finger positions, palm position, distance between positions, angles between positions	MLP	90
Proposed work	36 ASL	12 people × 1 set	Leave-one-subject-out cross-validation	Standard deviation of palm positions, hand palm curvature radius, distance between palm center and each fingertips, angle between two adjacent fingertips, distance between fingertips and each consecutive fingertips	DNN	93.81 (26 classes) 88.79 (36 classes)
